# Key Opportunities to Replace, Reduce, and Refine Regulatory Fish Acute Toxicity Tests

**DOI:** 10.1002/etc.4824

**Published:** 2020-08-24

**Authors:** Natalie Burden, Rachel Benstead, Kate Benyon, Mark Clook, Christopher Green, John Handley, Neil Harper, Samuel K. Maynard, Chris Mead, Audrey Pearson, Kathryn Ryder, Dave Sheahan, Roger van Egmond, James R. Wheeler, Thomas H. Hutchinson

**Affiliations:** ^1^ NC3Rs London United Kingdom; ^2^ Fera Science, Sand Hutton York United Kingdom; ^3^ Syngenta, Product Safety, Bracknell Berkshire United Kingdom; ^4^ Chemicals Regulation Division, Health and Safety Executive York United Kingdom; ^5^ Department for Environment, Food and Rural Affairs London United Kingdom; ^6^ Charnwood Molecular Nottingham United Kingdom; ^7^ Chemicals Regulation Division, Health and Safety Executive, Bootle Merseyside United Kingdom; ^8^ AstraZeneca, Eastbrook House Cambridge United Kingdom; ^9^ Batelle, Chelmsford Essex United Kingdom; ^10^ Environment Agency Wallingford Oxfordshire United Kingdom; ^11^ Home Office Dundee United Kingdom; ^12^ Cefas Fisheries Laboratory, Lowestoft Suffolk United Kingdom; ^13^ Unilever, Safety & Environmental Assurance Centre, Sharnbrook Bedford United Kingdom; ^14^ Shell Health, Shell International The Hague The Netherlands; ^15^ University of Plymouth, Plymouth Devon United Kingdom

**Keywords:** Aquatic toxicology, Ecotoxicology, Hazard/risk assessment, Chemical regulation

## Abstract

Fish acute toxicity tests are conducted as part of regulatory hazard identification and risk‐assessment packages for industrial chemicals and plant protection products. The aim of these tests is to determine the concentration which would be lethal to 50% of the animals treated. These tests are therefore associated with suffering in the test animals, and Organisation for Economic Co‐operation and Development test guideline 203 (fish, acute toxicity) studies are the most widely conducted regulatory vertebrate ecotoxicology tests for prospective chemical safety assessment. There is great scope to apply the 3Rs principles—the reduction, refinement, and replacement of animals—in this area of testing. An expert ecotoxicology working group, led by the UK National Centre for the Replacement, Refinement and Reduction of Animals in Research, including members from government, academia, and industry, reviewed global fish acute test data requirements for the major chemical sectors. The present study highlights ongoing initiatives and provides an overview of the key challenges and opportunities associated with replacing, reducing, and/or refining fish acute toxicity studies—without compromising environmental protection. *Environ Toxicol Chem* 2020;39:2076–2089. © 2020 The Authors. *Environmental Toxicology and Chemistry* published by Wiley Periodicals LLC on behalf of SETAC.

## INTRODUCTION

**Figure 1:**
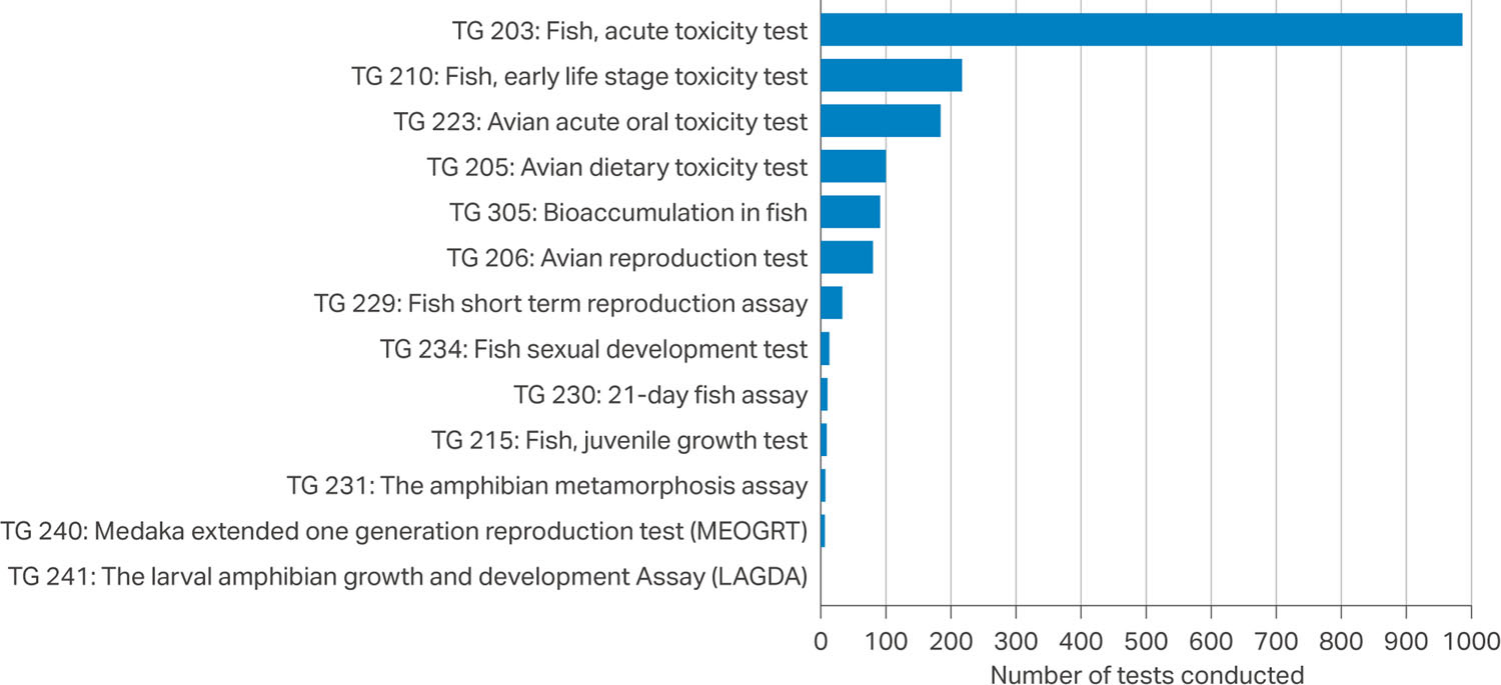
Number of Organisation for Economic Co‐operation and Development test guideline vertebrate ecotoxicology studies conducted across 15 contract research organizations from 2014 through 2017. More information on this survey can be found in Burden et al. (2017). Fish acute toxicity studies (in accordance with Organisation for Economic Co‐operation and Development 2019a) were by far the most frequently conducted. TG = test guideline.

Originally employed in the nineteenth century as a forensic tool for investigating fish kills from effluent pollution, fish acute toxicity tests have become a core requirement for prospective safety assessment under many global regulatory frameworks for manufactured chemicals, including plant protection products (PPPs) and industrial chemicals (Table 1; Hunn 1989; Hutchinson et al. 2016). They are used to determine the lethal concentration of a substance that causes death in 50% of the test population (LC50) during short‐term exposure—over hours or days. Fish acute toxicity studies are now the most frequently conducted vertebrate ecotoxicology tests (Figure 1; Burden et al. 2017) and one of the very few standardized vertebrate ecotoxicity tests where death is the intended endpoint—often causing significant suffering to test animals. This prompts the question: What are the key opportunities to apply the 3Rs principles—reduction, refinement, and replacement (see Table 2)—to the fish acute toxicity test in a regulatory setting?

**Table 1:** Fish acute toxicity data requirements by sector and global region, based on the Fish Toxicity Testing Framework (Organisation for Economic Co‐operation and Development 2014) and Maynard et al. (2017)^a^

Industry sector	Region	Example legislation	Acute in vivo test required for active substances (yes/no)	Species required/recommended, and product/formulation testing requirements
Plant protection products	EU	European Commission (2013)	Yes	Always required for rainbow trout (*Oncorhynchus mykiss*, cold‐freshwater species) Testing of products and ingredients, where it cannot be predicted based on ingredients, per Regulation 284/2013
	North America	US Federal Insecticide, Fungicide and Rodenticide Act, Canadian Plant Protection Product Active Substances (Pest Management Regulatory Agency)	Yes	Cold‐ and warm‐water freshwater species; 1 saltwater fish species, dependent on use Testing of ingredients only
	Latin America	Brazil (IBAMA): Portaria Normativa IBAMA no. 84, de 15 de outubro de 1996	Yes	Cold freshwater, warm freshwater dependent on country Testing of products and ingredients
	Asia Pacific	Japanese Agricultural Chemicals Regulation; China, Ministry of Agriculture and Rural Affairs: e.g., Measures on the Management of Pesticide Registration and Measures for the Administration of Pesticide Labels and Manuals, and the Data Requirements on Pesticide Registration (defines data required for submissions); Republic of Korea, Ministry of Agriculture, Food and Rural Affairs: Pesticide Control Act Enforcement Decree of the Pesticide Control Act	Yes	In‐country testing may be required. For example, only test reports obtained from qualified testing facilities can be recognized for registration review in China. Unilateral acceptance of data generated following OECD TGs to GLP from overseas laboratories is not possible unless there is a multilateral acceptance of data agreement with China. Local fish species may be required to be tested; e.g., in Bangladesh 2 local species are required to be tested in‐country for formulations. Some countries require testing of specific species for specific uses; e.g., in South Korea loach is required for paddy uses. Testing of products and ingredients can be required
	Notes	Estimated total number of different species tested in practice for global registration of an active substance = 4 (rainbow trout in the European Union, fathead minnow and sheepshead minnow in the United States, and carp in Japan). Can be higher if multiple Asian countries require specific native species. Multiple additional studies may be required, depending on the number of products requiring formulation testing.		
Industrial chemicals	EU	European Commission (2006)	Yes, if manufactured or imported at >10 tonnes/yr	Not specified; includes reference to species included in OECD TG 203.
	North America	US industrial chemicals Toxic Substances Control Act for New Substances (premanufacture notices) and Existing Substances	Can be requested following modeling outcome; can be requested following data review and risk determination	Cold‐ and warm‐freshwater species; if a marine or estuarine system may be affected, saltwater species also generally required.
	Asia Pacific	Japanese Chemical Substances Control Law; Australian Industrial Chemicals (Notification and Assessment) Act 1989; China New Substance Registration—China REACH—required by China's Ministry of Ecology and Environment	Yes	Cold freshwater or warm freshwater, dependent on country; in‐country testing may be required (e.g. China). State Environmental Protection Administration, China, Guidelines for the testing of chemicals, no. 203 fish acute toxicity test (2004) requires freshwater zebrafish (*Brachydanio rerio*)
Biocides	EU	EU Biocidal Products Regulation (Regulation EU 528/2012)	Yes	One freshwater (+marine species, if relevant) Testing of products and ingredients may be required in some circumstances depending on the use pattern, relative sensitivity of other taxa compared to fish, and if the risk cannot be predicted/resolved based on the ingredients. Exemptions can apply to active substance data, if 1) valid chronic (long‐term) fish toxicity data are available or 2) in some rare cases, if negligible exposure is expected (attributable to the use pattern or properties of the active substance)
	North America		Yes	Cold freshwater, warm freshwater, marine; requirements can potentially be reduced dependent on use or expected exposure Testing of ingredients
	Asia Pacific		Yes	Cold freshwater; requirements can potentially be reduced dependent on use or expected exposure or country Testing of products and ingredients
	Notes	For some product categories the data are already available from plant protection product registrations (e.g., certain fungicides and insecticides).		
Human pharmaceuticals	EU	EU Human Pharmaceuticals (Regulation EC 726/2004)	No; considered not relevant because of long‐term, low‐level exposure	n/a
	North America	US Food and Drug Administration Center for Drug Evaluation and Research	Yes; action limit at expected environmental concentration >100 ng/L (if not an endocrine‐disrupting compound), then a tiered approach if *Daphnia* or algae risk quotient <1000	Not specified
	Notes	For most global submissions, chronic testing conducted for EU submissions enables skipping of acute tiers of US requirements; see Supplemental Data 1.		
Cosmetics	EU		No, although information on fish may be required on ingredients covered under REACH (>10 tonnes/yr); see Notes	
	Rest of world		Country‐specific	Dependent on country; requirement in China for ingredients imported >1 tonne/yr to test with a native species
	Notes	Regulation (EC) No 1223/2009 of the European Parliament and of the Council of 30 November 2009 on cosmetic products sets out a testing ban—prohibition on testing finished cosmetic products and cosmetic ingredients on animals and a marketing ban, prohibition on marketing finished cosmetic products and ingredients in the European Union which were tested on animals.		

^a^ Notably, testing in multiple species is required to register an agrochemical globally, whereas for human pharmaceuticals and cosmetics, minimal or no testing is required.

GLP = good laboratory practice; IBAMA = Brazilian Institute of the Environment and Renewable Natural Resources; n/a = not applicable; OECD = Organisation for Economic Co‐operation and Development; TG = test guideline.

**Table 2:** Definitions of the 3Rs^a^

	Standard	Contemporary
Replacement	Methods which avoid or replace the use of animals	Accelerating the development and use of models and tools, based on the latest science and technologies, to address important scientific questions without the use of animals
Reduction	Methods which minimize the number of animals used per experiment	Appropriately designed and analyzed animal experiments that are robust and reproducible and truly add to the knowledge base
Refinement	Methods which minimize animal suffering and improve welfare	Advancing animal welfare by exploiting the latest in vivo technologies and by improving understanding of the impact of welfare on scientific outcomes

^a^ From National Centre for the Replacement, Refinement and Reduction of Animals in Research (n.d.).

Standardized protocols are followed when data from fish acute toxicity studies are generated to meet regulatory requirements, including the Organisation for Economic Cooperation and Development's (OECD's) test guideline 203 (Organisation for Economic Co‐operation and Development 2019a) and the US Environment Protection Agency's (USEPA's) test OCSPP 850.1075 (US Environment Protection Agency 2016a). Results are most often submitted along with data on acute toxicity to invertebrates and algae. For the prospective safety assessment of chemicals (the scope of the present study), information is used for hazard classification and labeling purposes and to assess risk to fish potentially exposed to substances following their use or discharge into the environment. It can also be used for nonregulatory purposes or for internal company decision‐making, such as molecule development and optimization, and to help select concentration ranges for testing in longer‐term (chronic) toxicity or bioaccumulation studies. Regulatory testing is also conducted for the purposes of measuring water quality (e.g., effluent assessments), although this aspect is not the focus of the present study.

Numerous ethical, scientific, business, and legislative drivers are compelling a shift in the current safety‐assessment paradigm away from established approaches, which largely rely on the generation of data from whole‐animal (in vivo) studies (Burden et al. 2015). Many legislations governing chemical safety assessment, particularly in Europe, demand that vertebrate animal tests are conducted as a last resort, that nonanimal methods are used where possible, and that existing data are shared (e.g., European Commission 2006, 2009a, 2009b). Regional bans on the marketing of cosmetics containing new ingredients tested on animals for human safety assessment purposes have also come into force (e.g., Europe, India, Israel and Brazil; European Commission 2009b; Burden, et al. 2016a; Table 1). In 2019, a bold move was made by the USEPA—the body responsible for regulating industrial chemicals and PPPs in the United States—by announcing its plan to eliminate requests for mammalian safety tests by 2035 (US Environment Protection Agency 2019). Efforts have already started to reduce and refine mammalian acute toxicity tests where they remain mandatory, for example, through activities to modernize the USEPA's PPP data requirements (US Environment Protection Agency 2017; Prior et al. 2019), deletion of the OECD's acute oral toxicity test (test guideline 401; Organisation for Economic Co‐operation and Development 1987) in 2002 specifically because of animal welfare concerns, and the recent adoption of OECD test guideline 433 (Organisation for Economic Co‐operation and Development 2018) to increase the use of more humane endpoints in place of death in acute inhalation studies (Sewell et al. 2015, 2018). In nonmammalian toxicology there have been similar efforts to improve the welfare implications of avian acute toxicity testing, through introduction and adoption of OECD test guideline 223 (avian acute oral toxicity test) (Organisation for Economic Co‐operation and Development 2016a). This test uses fewer animals than the alternative method (OCSPP 850.2100) (US Environmental Protection Agency 2012) and provides refinements, for example, through the option for single‐dose limit testing where toxicity is likely to be low (Edwards et al. 2017).

At the same time, there have been significant developments in biomedical science which will impact how (eco)toxicology and safety assessments are conducted in the future, together with a greater focus on environmentally realistic exposure scenarios. This includes the development of more sophisticated and physiologically relevant cell‐based approaches and the application of computational chemistry and mathematical modeling to biological systems. There are many current efforts focused on developing frameworks to allow data from these newer, nonanimal approaches to be used in place of traditional in vivo results when making chemical safety decisions (e.g., Organisation for Economic Co‐operation and Development 2016b). There may also be a need to rethink how decisions are arrived at as a result of changing environmental factors (e.g., climate change) and the emergence and evolution of new substance types (e.g., nanomaterials). Now is the time to turn the focus on harnessing the opportunities to improve the science underlying fish acute toxicity assessments, while at the same time enabling traditional in vivo approaches to be waived or replaced and, where still absolutely necessary, to reduce or refine in vivo studies to use fewer animals and/or induce less suffering—across all geographic regions and industry sectors.

The present study has been prepared by the UK's National Centre for the Replacement, Refinement and Reduction of Animals in Research (NC3Rs) ecotoxicology expert working group, following several discussions around the potential to apply the 3Rs in fish acute toxicity testing. Firstly, in the section *Current State of the Science, Testing Frameworks, and Key 3Rs Opportunities*, we identify pertinent areas for applying the 3Rs—including novel developments and existing opportunities with scope for wider uptake across a range of regulatory sectors. These are then summarized in the section *Harnessing the Opportunities*, with specific examples of data analysis or other work needed to clarify some of the uncertainties and enable definitive improvements to fish acute toxicity testing and assessment frameworks.

## CURRENT STATE OF THE SCIENCE, TESTING FRAMEWORKS, AND KEY 3Rs OPPORTUNITIES

### Question 1: Are new fish acute toxicity data always needed?

The first aspects to consider before carrying out any in vivo test should be whether the data are relevant to the regulatory and scientific question and whether data already exist that could answer that question.

#### Are fish acute toxicity data needed at all? Exposure considerations

In the “real world,” fish may rarely be exposed to chemicals at high concentrations and/or within the time frame of an acute toxicity test (which may better reflect an accidental spill, inadequately treated discharge, or improper disposal scenario). There may be limited uptake by fish if a substance has certain physicochemical properties such as low water solubility or a high melting point (Mayer and Reichenberg 2006). Some substances are readily biodegradable (or highly labile), and exposure in the environment may be very low following removal in wastewater‐treatment plants, in regions where these exist. These properties can often be readily identified, particularly if the substance will not dissolve (e.g., solid waxes), or by using modeling and analytical means (e.g., Thomas et al. 2015). Evidence of no or very low uptake may justify waiving acute in vivo tests in some regulatory regimes. Negligible release of a substance into the environment would also lead to no or very low environmental exposure (in the absence of spill events), or release may take place over longer durations. In this case, results from chronic tests may be more relevant. Human and veterinary medicinal products are most likely to be present over the long term at low levels because they mainly enter the environment following patient use. Therefore, acute testing for European pharmaceutical registrations is only triggered when a predicted environmental concentration reaches a threshold level, which has been based on historical acute environmental toxicology data (Table 1; European Medicines Agency 2006). This approach is considered highly protective (Gunnarsson et al. 2019) and has contributed in part to the practical removal of the requirement for fish acute toxicity tests for pharmaceuticals. The European Union's Registration, Evaluation, Authorisation and Restriction of Chemicals (REACH) regulation for industrial chemicals also uses a pseudo‐exposure‐driven approach because only substances produced or imported over 10 tonnes/yr require fish acute toxicity data (Table 1). These “exposure‐driven” approaches are lacking in other legislation and regulations including PPPs, where testing is in practice mandatory irrespective of environmental exposure. A stronger focus on exposure‐driven approaches has emerged in human health safety assessment (National Research Council 2012; Sewell et al. 2017a) and is equally applicable and beneficial to ecotoxicology. Wider implementation of exposure rather than hazard‐based assessments could greatly reduce the number of vertebrate studies. Retrospective analysis of existing data would help to determine suitable exposure triggers for testing (per Gunnarsson et al. 2019). Evidence of low or negligible exposure could at the very least help prioritize vertebrate testing, if not all ecotoxicity testing. Future modeling efforts should consider how chemical property and fate and effect data may be effectively used to predict impacts from variable exposure scenarios such as chemical spills.

#### Do data already exist to answer the scientific question? Exploiting options to waive new experimental tests

Existing data on the chemical or information on similar chemicals may be available to conduct safety assessments without the need for new data. The first step is to collate and review all available ecotoxicity, fate, and physicochemical data. Fish acute data may not be needed to characterize environmental risk if fish are not always the most sensitive taxa for acute effects, as is the case for many pharmaceuticals, industrial chemicals, and PPPs (Weyers et al. 2000; Hutchinson et al. 2003; Jeram et al. 2005; Rawlings et al. 2019; Lillicrap et al. 2020). Although many regulatory regimes require ecotoxicity data across 3 trophic levels, conducting tests using nonvertebrate species (i.e., invertebrates and algae/aquatic plants) first may provide sufficient information for safety assessment when considered with other data, particularly if the chemical falls into a class where potential toxic effects are well known (see section *Grouping and read‐across approaches*). In chronic assessments of antibiotics, fish testing is now not required because of the known lack of sensitivity (Baumann et al. 2015; Brandt et al. 2015; Le Page et al. 2017; Committee for Medicinal Products for Human Use 2018). Better understanding of evolutionary target conservation (i.e., biological similarity) across ecological taxa would be useful to determine whether fish data are relevant. This has started to be explored for the chronic effects of pharmaceuticals using tools such as ECOdrug (n.d.; Gunnarsson et al. 2019).

Existing fish in vivo data, even with limitations or restrictions, may be sufficient for decision‐making. Where regulations allow, studies that do not adhere to a good laboratory practice (GLP) framework or open literature studies may be used to consider relative trophic‐level sensitivity or support assumptions of low toxicity. All data should be evaluated in terms of reliability and relevance (e.g., Moermond et al. 2016), in a way applicable to the regulatory framework (Martin et al. 2019). Existing reliable data on chronic fish toxicity may be relevant to acute effects or support the assumption that fish are less sensitive than other aquatic species. The European Union Biocidal Products Regulation guidance already indicates that if a valid chronic study on fish is available, acute data are not needed (European Chemicals Agency 2018). This can also be the case on a substance registration–specific basis for industrial chemicals under the European Union's REACH regulation (Annex VIII; European Commission 2006). Existing data could also provide justification for less animal‐intensive or refined acute toxicity studies (e.g., application of an in vivo fish limit test, fish embryo acute toxicity test [FET], or fish in vitro cell line assay; discussed in the section *Embryo and in vitro assays*).

#### Grouping and read‐across approaches

Existing data on substances that fall within the same “group” as the test substance can be useful. Grouping is based on, for example, structural similarities or the sharing of common metabolic pathways. These properties can be used to predict likely physicochemical, fate, and ecotoxicity profiles, the principle being that the group of substances will exert similar effects. For example, the PETROTOX model has been developed to perform aquatic hazard assessment of petroleum substances based on substance composition (Redman et al. 2017). Here, hydrocarbons of similar structure and size within a petroleum substance are grouped together because they are known to behave similarly in terms of environmental distribution and fate (Concawe 1996). Toxicity or environmental risk limits of these complex mixtures can then be calculated without the need for all components to be tested experimentally.

A similar approach involves read‐across of experimental data from one substance (source) to another (target); for an example, see Supplemental Data 2. It is already possible to use read‐across for classification and labeling purposes in the European Union if fish acute data are not available (European Chemicals Agency 2017a). Significant documentation is required to justify the validity of grouping or source data and demonstrate the relevance of the grouping or read‐across approach, as set out, for example, by the European Chemicals Agency (2008, 2017b), and the OECD (Organisation for Economic Co‐operation and Development 2017a). One drawback with read‐across approaches is that they cannot be used where there are no existing experimental toxicity data for a new chemical class to read‐across from. In addition, when considering read‐across for risk management, the uncertainties associated with it may prove unfavorable.

### Question 2: Can standard fish acute toxicity studies be replaced?

Information on fish acute toxicity will often be necessary to enable safety assessment and environmental protection. But must this always come from traditional in vivo studies? Wording in the recently revised test guideline 203 (Organisation for Economic Co‐operation and Development 2019a) suggests that existing in vivo data as well as data from alternative approaches should be considered prior to conducting a full test guideline 203 test. Several alternative/replacement approaches which could provide useful data exist or are under development. These include predictive computational tools or in silico methods, such as quantitative structure–activity relationship models (QSARs), or experimental techniques which do not require the use of protected animals (defined as vertebrate animals used for scientific purposes, protected under legislation such as European Union Directive 2010/63 [European Commission 2010], including early life‐stage embryos).

#### Computational models

A QSAR is a statistical model used to relate a substance's structure to toxic outcomes. Such models have started to be used in a regulatory context to predict fish acute toxicity. The majority of QSARs conducted for lower‐tier endpoints under the European Union REACH regulation were for this purpose (European Chemicals Agency 2017c), and regulatory agencies themselves have developed models, such as the ECOlogical Structure Activity Relationships (ECOSAR) Class Program provided by the USEPA. The ECOSAR program was used to assess the utility of QSARs in predicting fish acute toxicity of PPP metabolites and was shown to be a robust approach worthy of further investigation, with potential for use in regulatory decision‐making (Burden et al. 2016b). There is already one example in Europe where ECOSAR was used to estimate the toxicity of metabolites of the biocidal active substance imiprothrin (European Chemicals Agency 2018). Although this is promising, substances can only be assessed using QSARs if they fall into one of the chemical classes for which the model has been validated. Their use for chemicals with novel chemistries is limited until experimental data are available and subsequently incorporated into the models. Further, significant documentation describing the QSAR inputs and model is required to justify the prediction before QSAR endpoints will be considered in a regulatory setting (e.g., see European Chemicals Agency 2008).

Other computational models that could prove useful include the USEPA's Interspecies Correlation Estimation platform (US Environmental Protection Agency 2016b). These models estimate the acute toxicity of a chemical to a species, genus, or family with no test data (the predicted taxon) from the known toxicity of the chemical to a species with test data (the surrogate species). Invertebrate data could therefore be used to make predictions of fish acute toxicity without conducting a fish test, although further work will be needed to support such extrapolations for regulatory acceptance. Alternatively, where experimental data are available for one fish species, they could be used to 1) extrapolate to other fish species (particularly valuable for sectors where multiple species currently require testing; see section *Embryo and in vitro assays* and Table 1) and/or 2) aid in evaluation of interspecies variability for refinement of risk assessments (European Food Safety Authority 2013). The Sequence Alignment to Predict Across Species Susceptibility tool is another promising approach which could in future be used in a regulatory context to support extrapolation of toxicity information across species (LaLone et al. 2016).

#### *Embryo and in vitro* *assays*

Prior to embryos becoming free‐feeding, they are considered to be incapable of experiencing pain, distress, suffering, or lasting harm (European Food Safety Authority 2005) and in many regions are not protected under animal welfare legislation in the same way as juvenile and adult fish (e.g., European Commission 2010). Their use is therefore considered beneficial from a 3Rs perspective, and the potential for fish embryo assays such as the FET to be used in place of studies conducted at later life stages has been extensively investigated. A version of the FET is already used in Germany to assess the fish acute toxicity of effluents (International Organization for Standardization 2007; Bundesministerium für Umwelt, Naturschutz und nukleare Sicherheit 2009; Lillicrap et al. 2016). For prospective chemical assessment there is an internationally validated method (OECD test guideline 236; Organisation for Economic Co‐operation and Development 2011a, 2011b, 2012, 2013). Results have correlated well with standard in vivo fish acute LC50 data for most chemicals tested (Braunbeck et al. 2005; Lammer et al. 2009; Knöbel et al. 2012; Belanger et al. 2013; European Commission 2014). Belanger et al. (2013) indicated that the high correlation observed between the FET and traditional fish acute toxicity studies was similar to that seen for traditional LC50 values generated between different species of fish, further supporting the robustness of the approach. The broad regulatory applicability of this assay has, however, been questioned (European Chemicals Agency 2016; see next section).

Great investment has also been made in developing in vitro cytotoxicity assays. The rainbow trout RTgill‐W1 cell line assay (International Organization for Standardization 2019; and OECD test guideline development in progress) demonstrates a good correlation with published in vivo LC50 endpoints for 35 chemicals with a range of properties and modes of action (Tanneberger et al. 2013). It has been shown to be robust and reproducible following a large‐scale international interlaboratory ring test (Fischer et al. 2019). The applicability domain of the assay has started to be characterized. For example, the acute toxicity of fragrance chemicals has been accurately demonstrated (Natsch et al. 2018), but the assay is known to be unsuitable for detecting acute toxicity to certain neurotoxicants such as those which act directly via brain tissue. Like many in vitro test systems, it has limited metabolic capacity (Tanneberger et al. 2013), although efforts are being made more widely to increase its metabolic competence and utility (e.g., Luckert et al. 2017).

### Replacement opportunity: Applying weight‐of‐evidence approaches

By nature, whole‐organism tests incorporate biological complexity; and in reality, no single nonanimal or alternative method can be used in isolation to replace this. It is natural for registrants and assessors to continue to use the “tried and trusted” (and legally mandated) methods such as in vivo fish acute toxicity tests, with which they are most familiar and for which data interpretation is relatively straightforward. There is still a need to build confidence in the use and interpretation of data from alternative methods despite them being validated to a high standard—greater than that of many of the classical in vivo test guidelines.

Despite the availability of a comprehensive data set, FET data are still not considered by regulatory authorities as a direct alternative to standard fish acute toxicity data, with the European Chemicals Agency citing that “a lack of quality data makes it challenging to conclude on several aspects of the applicability domain” (2016). The FET and other alternative data will gain wider acceptance if data from various methods are considered in combination—as part of so‐called weight‐of‐evidence approaches (defined in European Chemicals Agency 2017b; also see European Chemicals Agency 2017d; Organisation for Economic Co‐operation and Development 2019b). Examples of the successful use of combination approaches have just started to emerge under REACH (Supplemental Data 2). The practical implementation of the FET within a combination approach has started to be explored (Lillicrap et al. 2020; HUGIN SWiFT n.d.). A current OECD project is also examining the potential to incorporate the FET into the threshold approach (discussed in the section *Refinement and reduction opportunity: Reducing the number of test groups*), as part of an integrated approach to testing and assessment (IATA) to minimize testing on juvenile or adult fish (unpublished data). Data from assays such as the RT‐gill cell line may also be incorporated into the IATA. Once published, the IATA approach would still need to be adopted within regulatory frameworks before it could be applied in practice. The key consideration before this happens is whether the combination approach can sufficiently protect the environment from the potential impacts of chemicals—no attempts to our knowledge have been made to assess how well even traditional methods achieve this. For sectors such as the cosmetics industry, the option to fall back on in vivo study data no longer exists, at least in the assessment of human health effects. In a hypothetical situation where fish acute toxicity tests were no longer an option across all sectors, what would the new data package look like? In practice, changes in safety‐assessment practices will realistically need to be driven top‐down by legislative and regulatory change.

### Question 3: Can standard studies be reduced and refined?

In the shorter term, where standard studies remain absolutely necessary, key opportunity areas center around 1) reducing the number of studies conducted and the number of animals used within studies and 2) refining the tests to decrease the suffering experienced by the test animals.

### Reduction opportunity: Harmonizing global test guidelines and regulatory requirements

Because most new substances/products are developed for global use, data packages are usually generated to meet the data requirements of all the regions and countries for which registration and marketing are intended (see Table 1). The most commonly used fish acute toxicity guideline for prospective assessments globally is OECD test guideline 203 (Figure 1). Under the Mutual Acceptance of Data (MAD) agreement, when an OECD test guideline is conducted in one adherent country under GLP, the data generated will be accepted in other countries adhering to MAD—with the aim of avoiding test duplication. Recent estimates predicted that MAD reduces the number of animals needed in testing new industrial chemicals by 32 702 (Organisation for Economic Co‐operation and Development 2019c). Despite adhering to MAD, some geographical regions specify a preference for other standardized methods (e.g., US Environmental Protection Agency 2016a), which can have marked differences in study designs—translating to variations in the overall number of fish used (see Supplemental Data 3; n.b., part of the rationale for the last revision of test guideline 203 was to improve harmonization with the US guideline). There are some examples of non‐OECD member countries adopting OECD test guidelines or having their own but virtually identical protocols which specify differences such as test species (Table 1). It can also be the case, such as in China (ChemicalWatch 2019), that testing must be conducted within the country of registration using local species. There is no guarantee that data generated in non‐OECD member countries will be accepted in other regions.

#### Reducing the number of fish species used in PPP testing

The acute toxicity testing of PPPs is a prime example where differing regional requirements drive high fish use because 4 or more different species may be required for truly global registrations (Table 1). The test species required in different regions is guided by a range of considerations, including the intended application of the chemical; differing environmental exposure scenario(s) (for example, likely release into fresh or saltwater); commercially or ecologically important species in a given region; or special conservation measures for certain groups of endangered freshwater, estuarine, or marine species (see Table 1). It may be the case, however, that testing of the same substance in multiple fish species is not necessary if they all share the same key biological pathways resulting in adverse effects (Ankley et al. 2010; Groh and Tollefsen 2015). Understanding the similarities and differences in xenobiotic metabolism in different fish species is also important here (Kleinow et al. 1987; Nichols et al. 2007).

The key needs are to 1) better understand and/or 2) account for differences in species sensitivity. In reference to point 1, some investigations have been made to explore differences in sensitivity to acute effects in different fish species. Several papers suggest that rainbow trout (*Oncorhynchus mykiss*) is the most sensitive species across a diverse range of chemicals (Dyer et al. 1997, 2006; Lammer et al. 2009), and the authors question the need for separate testing of tropical fish species. An analysis conducted by the European Food Safety Agency (2006) also showed that rainbow trout was generally the most sensitive species (though the comparator species was not the same for each substance, and caution should be exercised in the interpretation of these data). The sensitivity of saltwater versus freshwater fish has also been compared (Hutchinson et al. 1998; Leung et al. 2001; Wheeler et al. 2002, 2014; Maltby et al. 2005) with this literature, suggesting that saltwater and freshwater fish are similarly sensitive. In line with this there has been a rationalization of testing requirements in certain regions. For PPP active substances in Europe only a fish acute toxicity test conducted using rainbow trout is now required (European Commission 2013), where previously a warm‐water species was also mandatory (European Commission 1991). The USEPA in collaboration with the National Toxicology Program's Interagency Center for the Evaluation of Alternative Toxicological Methods is currently conducting a retrospective analysis project to assess whether current US regulatory requirements for the testing of warm‐ and cold‐freshwater plus estuarine/marine fish can be reduced to 2 or even one species (National Toxicology Program 2020). To point 2, part of the reason for applying “assessment factors” to the lowest (eco)toxicity value in the derivation of predicted‐no‐effect concentration is to account for variability in species sensitivity. Where species sensitivity differences are not anticipated, additional species testing should not be required because the degree of sensitivity difference should in theory fall within the variation accounted for by the assessment factor. Greater consideration of likely exposure scenarios can also reduce the number of species used for testing (as discussed in the section *Reducing the number of fish species in PPP testing*). This is currently an option in the United States; if, for example, a PPP is not going to be applied in regions adjacent to mangroves, it may be possible to waive testing in a saltwater species.

Clearly, individual regional provisions that reduce the number of fish species tested are likely to have a limited impact on the 3Rs. Global harmonization of vertebrate regulatory requirements is needed, but this will be a difficult and slow process because local legal and societal needs must be considered.

#### Reducing the need for formulation testing

For some substance types, likely environmental exposure scenarios mean that fish acute testing of finished/formulated products is not a standard requirement (Table 1)—for example, human medicines are most likely to enter the environment following patient use (German Advisory Council on the Environment 2007) having undergone significant modification from the administered formulated product. However, PPP active substances may be used in differing formulations with, for example, slight differences in the adjuvants/solvents used; and under some regional requirements each mixture must be assessed independently (Table 1). This often requires that in vivo data be generated for each formulation. This is despite evidence that tests conducted with the active substance could potentially predict the formulation toxicity (Schmuck et al. 1994) and that most formulations are not expected to exhibit more than additive toxicity compared with their constituent active substance components (Creton et al. 2014). Even where there are provisions to waive testing of “similar” formulations, in practice this can be challenging. For example, the European Union PPP regulation (European Commission 2009a) states that fish acute toxicity testing shall be performed where the acute toxicity of the PPP cannot be predicted on the basis of the data for the active substance and extrapolation on the basis of available data for a similar PPP is not possible. However, there is a lack of agreed guidance on how to robustly demonstrate that a preparation is sufficiently similar or that toxicity can be reliably predicted. There are already ongoing efforts to decrease the need for mammalian acute toxicity testing of formulations for human health assessments. This includes a program by the USEPA to evaluate the ability of the Globally Harmonised System of Classification and Labelling of Chemicals dose additives mixtures equation to predict acute toxicity categories for PPP formulations/products (US Environmental Protection Agency 2017; note for classification and labeling of industrial chemical mixtures in Europe, hazard classification is already calculated on the basis of the proportional ecotoxicity of components and their percentage of concentrations). More sophisticated platforms are also under development for predicting the toxicity of PPP formulations (e.g., see National Centre for the Replacement, Refinement and Reduction of Animals in Research 2018). Similar approaches for fish acute toxicity data, particularly if the purpose of data generation is solely hazard identification and classification, may be viable.

#### Refinement and reduction opportunity: Reducing the number of test groups

Limit tests are performed with a single concentration of 100 mg/L (or limit of solubility) using 7 or more fish. In the absence of mortality, there is at least 99% confidence that the LC50 is greater than the tested concentration—considered nontoxic to fish for most safety‐assessment purposes (Organisation for Economic Co‐operation and Development 2019a). Another way to reduce fish numbers is through the “threshold approach”—where an initial test is carried out in fish at one concentration selected based on the results of *Daphnia* and algae toxicity tests. The full response concentration series is only triggered if mortality is observed at this threshold concentration. Retrospective data analysis has demonstrated that a reduction of between approximately 38 and 73% of fish use could be achieved using a threshold approach, depending on the sector (Hutchinson et al. 2003; Jeram et al. 2005; Creton et al. 2014). It can also be viewed as a refinement because high doses need not be tested so often. The approach works well for risk assessments which consider the aquatic compartment as a whole, where endpoints are combined to derive an assessment that is applied to the lowest value from the different taxa. However, its application can be complicated for regulations that require risk assessments for individual taxa because varying assessment factors and refinements may be needed (Creton et al. 2014). It has been suggested that FET data could be used in concentration range‐finding (Rufli and Springer 2011; Rawlings et al. 2019; Organisation for Economic Co‐operation and Development 2019a), although the logistics of incorporating such testing strategies into commercial operations would be challenging (related to scheduling, analytical verification, etc., and the limited number of laboratories offering FET as a routine assay currently because of the lack of regulatory need and thus industry demand). It is unclear how widely threshold approaches are currently being applied in practice.

Test substances often require the use of solvents to aid dissolution, and typically a solvent control group is included in the study in addition to the dilution water control. Following the recent OECD test guideline 203 revision, there is now the option to omit the dilution water control group when a solvent is used, decreasing the number of fish used by 7 per test (Organisation for Economic Co‐operation and Development 2019a). The OECD has an ongoing project to determine the statistical power of test guideline 203 studies with solely a solvent control, and results indicate that there is no need to include a water control (unpublished data). It is not clear yet how widely such an approach will be accepted across different regulatory authorities/jurisdictions, particularly those which prefer non‐OECD test guidelines. The recently updated OECD guidance document 23 on aqueous‐phase aquatic toxicity testing of difficult test chemicals (Organisation for Economic Co‐operation and Development 2019d) includes revisions to reduce occasions when solvents need to be used for poorly water‐soluble substances. This includes better use of different generator systems for dosing, including passive dosing methods. Where solvent use is avoided, the solvent control group is not necessary. However, it should be acknowledged that there are situations where solvent‐assisted test item delivery is required to achieve appropriate exposure and ensure that a valid test is performed (Green and Wheeler 2013). In such cases, it is better to have employed a solvent control and deliver a robust study that does not subsequently need repeating. The provision of improved technical guidance such as the updated guidance document 23 can also help to reduce the need to repeat studies by supporting the fulfillment of recommendations or validity criteria within test guidelines (Burden et al. 2017).

### Refinement opportunity: Using humane/early endpoints in place of lethality

Early euthanasia of moribund fish is often practiced in Europe and Canada (with European Union Directive 2010/63/EU stating that death should be “substituted by more humane endpoints using clinical signs that determine the impending death” [European Commission 2010]). Humane endpoints are defined by the OECD as “the earliest indicator in an animal experiment of severe pain, severe distress, suffering, or impending death” (Organisation for Economic Co‐operation and Development 2000). The routine, global use of “sublethal” humane endpoints in place of death would substantially reduce the degree of suffering experienced by test animals. It may also be considered more environmentally relevant and indicative of “ecological death”—in the wild these fish would not survive (e.g., because of predation). There is already a precedent in mammalian acute toxicity testing where “evident toxicity” is used as the endpoint in place of death (e.g., Organisation for Economic Co‐operation and Development 2002, 2017b, 2018; Sewell et al. 2015). “Evident toxicity” is defined as clear signs of toxicity without causing severe toxic effects or mortality, which predict that exposure to the next highest concentration will cause severe toxicity or death/moribundity in most animals. There is, however, no international consensus on which sublethal clinical signs define moribundity or are predictive of death in fish. Early termination of studies when fish are showing signs of “considerable suffering” is featured in other test guidelines, but these are designed to examine less severe endpoints, for example, OECD 240 (Organisation for Economic Co‐operation and Development 2015). The 2019 revision to OECD test guideline 203 requires the recording of certain sublethal clinical signs, with the option to record further/more detailed signs. The aim of collecting these data is to enable the development of detailed guidance on how to identify when individual fish should be humanely terminated before the end of the test. A UK Department for the Environment, Food and Rural Affairs–sponsored expert workshop was held in early 2020 with the aim of identifying the knowledge gaps impeding the standardized recording and reporting of sublethal clinical signs of toxicity in fish and ultimately moving away from using mortality as the endpoint in OECD test guideline 203. It is critical that the signs identified are genuinely predictive of death because the use of humane endpoints could affect experimental endpoint outcome by lowering LC50 values. Lower LC50 values could trigger unnecessary higher‐tier testing within the same risk assessment framework and lead to the use of more animals.

## HARNESSING THE OPPORTUNITIES

The present exploration of the current situation has identified opportunities for further work to reduce uncertainty in key areas and genuinely support greater application of the 3Rs. This will only occur if strong scientific evidence is provided which demonstrates that protection goals will not be compromised. Table 3 summarizes examples of how these opportunities could be harnessed, under the themes of accelerating the acceptance of alternative approaches, improving global harmonization of data requirements, and reducing/refining within mandatory in vivo studies.

**Table 3:** Summary of examples of how key 3Rs opportunities could be harnessed

Aim	Approach	Notes and considerations
Accelerating the acceptance of alternative approaches		
To build confidence in the predictive value of alternative approaches	Implementation of “safe haven” approaches (Sewell et al. 2017b) where registrants submit both standard fish acute toxicity data in parallel with packages which utilize multiple lines of evidence generated using nontraditional methods (cf. the International Council for Harmonisation of Technical Requirements for Pharmaceuticals for Human Use trial for new approaches to carcinogenicity assessment [European Medicines Agency 2016]).	Places a relatively high financial and resource burden on industry and requires cross‐industry alignment in the approaches applied. Regulatory agencies would need to be open to and encouraging of such an approach and enter into formal agreements to retrospectively assess and publish scenarios where the nonstandard approaches are acceptable.
	Increase proficiency/capacity to conduct nonstandard approaches in contract laboratories.	Currently challenging because of lack of regulatory need/industry demand. May be incentivized by provision of safe haven approaches.
	Collect case studies on registered chemicals where alternative information types have been generated/used successfully. This could involve a “blinded” comparison of regulatory decisions/outcomes based on traditional vs alternative data.	This would also support the definition of the applicability domain of alternative approaches (based, e.g., on chemical class or mode of action).
	Support regulatory bodies in interpretation of integrated approaches to testing and assessment–style assessments for the fish acute toxicity endpoint, drawing on the new tools available.	This would be aided by the provision of relevant and clear guidance.
Improving global harmonization of data requirements		
To definitively determine whether 1) multiple species in PPP and biocide testing and 2) formulation in addition to active substance testing is needed and, where appropriate, align global data requirements.	Conduct retrospective data analysis to determine the difference in interspecies sensitivity on a range of compounds encompassing a broad range of modes of action that have been tested on a range of fish species. This should allow conclusions to be made on whether there is a generally sensitive fish species on which the application of assessment factors would provide suitable levels of protection.	Such an analysis should, if possible, also consider whether there are any significant differences in marine, brackish, freshwater, cold‐water, or warm‐water species and whether there is a difference between classes or subclasses of fish. Publicly available databases that may be drawn from, such as the EnviroTox database (Connors et al. 2019) and the OECD's eChem portal.
	Conduct retrospective data analysis comparing the acute toxicity of a range of different formulation types with the same active substance tested in the same species, to determine whether there are any patterns and if any general rules can be established. Further exploration of the use of reliable prediction methods (e.g., machine learning techniques), particularly for hazard identification and classification where substances are assigned into broad categories.	Calculated formulation toxicity could be compared with existing in vivo formulation testing data to validate the calculation approach. Guidance would then be prepared to advise on when a preparation is sufficiently similar to the active substance, negating the need for new testing. Prediction approaches may also be applicable to assess the toxicity of metabolites/degradation products, particularly for PPPs. This has started to be explored using QSAR models (see *Question 2: Can standard fish acute toxicity studies be replaced?*; Burden et al. 2016).
Reducing/refining animal use in mandatory in vivo studies		
To increase the uptake of the threshold approach and use of humane endpoints in place of lethality.	Identify the extent of and barriers to uptake of the threshold approach within industry and regulatory bodies, and devise efforts to overcome these.	
	Establish who will be responsible for collating sublethal clinical signs data with the cooperation of industry (as owners of the data). Conduct data analysis (retrospectively and through prospective data collection) to determine which sublethal signs are relevant and predictive and could be used as the modified endpoint.	These aspects were the subject of discussion at the 2020 UK‐Defra‐funded workshop (manuscript in preparation). This will require consistency between laboratories regarding identification and recording of signs within individual fish, which may not be possible retrospectively. Critical that there is global agreement on the standard use and definition of humane endpoints so as not to undermine the mutual acceptance of data agreement if some countries continue to require the lethality endpoint (note the authors have not experienced this to be the case to date).

Defra = Department for Environment, Food and Rural Affairs; OECD = Organisation for Economic Co‐operation and Development; PPP = plant protection product; QSAR = quantitative structure–activity relationship.

## IN SUMMARY

There is undoubtedly value in the different industry and regulatory sectors coming together in this unique way to share experiences and explore how approaches applied in one sector may be applicable to another. A key example includes considering the wider relevance and application of exposure‐driven approaches. For some sectors, such as the cosmetics industry, generating in vivo data is already legally no longer an option in many regions to assess human health effects, and absolute replacements must be utilized to address safety concerns. Global harmonization of requirements is paramount in maximizing the 3Rs impact on regulatory change. Pertinent questions remain: 1) Can alternative approaches be used in existing or modified regulatory hazard and/or risk assessment schemes to achieve at least the same level of protection compared with current practices (and if the appropriate level of protection is not afforded, what would be required to ensure that it is)? 2) What is actually needed to address perceived data gaps? There are further scientific reasons for reconsidering the current paradigm, to meet the challenges that changing environmental landscapes and technologies will raise. Ultimately, change in practice needs to be driven by a “top‐down” approach which compels the community to think differently—in line with the recent commitment by the USEPA to eliminate mammalian safety testing by 2035. It is the perfect time to reflect on the current situation and consider how to make best use of the new techniques and approaches available, while still ensuring that testing is science‐driven and protective—to genuinely reduce the number of fish experiencing severe suffering in the quest to assess chemical‐induced acute toxicity.

## Supplemental Data

The Supplemental Data are available on the Wiley Online Library at https://doi.org/10.1002/etc.4824.

## Disclaimer

The views and statements expressed in the present study are those of the authors alone. The views or statements expressed in this publication do not necessarily represent the views of the organizations to which the authors are affiliated, and those organizations cannot accept any responsibility for such views or statements.

## Author Contributions Statement

N. Burden wrote the manuscript. R. Benstead, K. Benyon, M. Clook, C. Green, J. Handley, N. Harper, S.K. Maynard, C. Mead, A. Pearson, K. Ryder, D. Sheahan, R. van Egmond, J.R. Wheeler, and T.H. Hutchinson participated in discussions to inform the content and provided technical and editorial assistance.

## Supporting information

This article includes online‐only Supplemental Data.

Supporting information.

Supporting information.

Supporting information.

## Data Availability

Data, associated metadata, and calculation tools are available from the corresponding author (natalie.burden@nc3rs.org.uk).

## References

[etc4824-bib-0001] Ankley GT , Bennett RS , Erickson RJ , Hoff DJ , Hornung MW , Johnson RD , Mount DR , Nichols JW , Russom CL , Schmieder PK , Serrrano JA , Tietge JE , Villeneuve DL . 2010 Adverse outcome pathways: A conceptual framework to support ecotoxicology research and risk assessment. Environ Toxicol Chem 29:730–741. 20821501 10.1002/etc.34

[etc4824-bib-0002] Baumann M , Weiss K , Maletzki D , Schüssler W , Schudoma D , Kopf W , Kühnen U . 2015 Aquatic toxicity of the macrolide antibiotic clarithromycin and its metabolites. Chemosphere 120:192–198. 25051235 10.1016/j.chemosphere.2014.05.089

[etc4824-bib-0003] Belanger SE , Rawlings JM , Carr GJ . 2013 Use of fish embryo toxicity tests for the prediction of acute fish toxicity to chemicals. Environ Toxicol Chem 32:1768–1783. 23606235 10.1002/etc.2244

[etc4824-bib-0004] Brandt KK , Amézquita A , Backhaus T , Boxall A , Coors A , Heberer T , Lawrence JR , Lazorchak J , Schönfeld J , Snape JR , Zhu YG , Topp E . 2015 Ecotoxicological assessment of antibiotics: A call for improved consideration of microorganisms. Environ Int 85:189–205. 26411644 10.1016/j.envint.2015.09.013

[etc4824-bib-0005] Braunbeck T , Boettcher M , Hollert H , Kosmehl T , Lammer E , Leist E , Rudolf M , Seitz N . 2005 Towards an alternative for the acute fish LC(50) test in chemical assessment: The fish embryo toxicity test goes multi‐species—An update. ALTEX 22:87–102. 15953964

[etc4824-bib-0006] Bundesministerium für Umwelt, Naturschutz und nukleare Sicherheit . 2009 Gesetz zur ordnung des wasserhaushalts (wasserhaushaltsgesetz‐WHG) (Law on the order of the Water Board). Series 1, German Federal Law Publications, Berlin, Germany.

[etc4824-bib-0007] Burden N , Benstead R , Clook M , Doyle I , Edwards P , Maynard SK , Ryder K , Sheahan D , Whale G , van Egmond R , Wheeler JR , Hutchinson TH . 2016a Advancing the 3Rs in regulatory ecotoxicology: A pragmatic cross‐sector approach. Integr Environ Assess Manag 12:417–421. 26440537 10.1002/ieam.1703

[etc4824-bib-0008] Burden N , Gellatly N , Benstead R , Benyon K , Blickley TM , Clook M , Doyle I , Edwards P , Handley J , Katsiadaki I , Lillicrap A , Mead C , Ryder K , Salinas E , Wheeler J , Hutchinson TH . 2017 Reducing repetition of regulatory vertebrate ecotoxicology studies. Integr Environ Assess Manag 13:955–957. 28834369 10.1002/ieam.1934

[etc4824-bib-0009] Burden N , Maynard SK , Weltje L , Wheeler JR . 2016b The utility of QSARs in predicting acute fish toxicity of pesticide metabolites: A retrospective validation approach. Regul Toxicol Pharmacol 80:241–246. 27235557 10.1016/j.yrtph.2016.05.032

[etc4824-bib-0010] Burden N , Sewell F , Chapman K . 2015 Testing chemical safety: What is needed to ensure the widespread application of non‐animal approaches? PLoS Biol 13:e1002156. 26018957 10.1371/journal.pbio.1002156 PMC4446337

[etc4824-bib-0011] ChemicalWatch . 2019 China product stewardship regulation introduction and latest update, 31 July 2019. [cited 2020 June 16]. Available from: https://events.chemicalwatch.com/79370/china-product-stewardship-regulation-introduction-and-latest-update

[etc4824-bib-0012] Committee for Medicinal Products for Human Use . 2018. Guideline on the environmental risk assessment of medicinal products for human use—Revision 1. Draft. EMEA/CHMP/SWP/4447/00 Rev. 1 European Medicines Agency, London, UK.

[etc4824-bib-0013] Concawe . 1996. Environmental risk assessment of petroleum substances: The hydrocarbon block method. Report 96/52. Brussels, Belgium.

[etc4824-bib-0014] Connors KA , Beasley A , Barron MG , Belanger SE , Bonnell M , Brill JL , de Zwart D , Kienzler A , Krailler J , Otter R , Phillips JL , Embry MR . 2019 Creation of a curated aquatic toxicology database: EnviroTox. Environ Toxicol Chem 38:1062–1073. 30714190 10.1002/etc.4382 PMC6850623

[etc4824-bib-0015] Creton S , Clook M , Wheeler JR . 2014 Application of the threshold approach for acute fish toxicity testing to plant protection products: A proposed framework. Chemosphere 96:195–200. 24183622 10.1016/j.chemosphere.2013.10.015

[etc4824-bib-0016] Dyer SD , Belanger SE , Carr GJ . 1997 An initial evaluation of the use of Euro/North American fish species for tropical effects assessments. Chemosphere 35:2767–2781.

[etc4824-bib-0017] Dyer SD , Versteeg DJ , Belanger SE , Chaney JG , Mayer FL . 2006 Interspecies correlation estimates predict protective environmental concentrations. Environ Sci Technol 40:3102–3111. 16719118 10.1021/es051738p

[etc4824-bib-0018] ECOdrug . n.d. University of Exeter, Exeter, Devon, UK. [cited 2020 June 18]. Available from: www.ecodrug.org

[etc4824-bib-0019] Edwards PJ , Leopold A , Beavers JB , Springer TA , Chapman P , Maynard SK , Hubbard P . 2017 More for less: Analysis of the performance of avian acute oral guideline OECD 223 from empirical data. Integr Environ Assess Manag 13:906–914. 28316137 10.1002/ieam.1930

[etc4824-bib-0020] European Chemicals Agency . 2008. Guidance on information requirements and chemical safety assessment. Chapter R.6: QSARs and grouping of chemicals. Helsinki, Finland.

[etc4824-bib-0021] European Chemicals Agency . 2016. Analysis of the relevance and adequateness of using fish embryo acute toxicity (FET) test guidance (OECD 236) to fulfil the information requirements and addressing concerns under REACH. Helsinki, Finland.

[etc4824-bib-0022] European Chemicals Agency . 2017a. Guidance on the application of the CLP criteria guidance to regulation (EC) no 1272/2008 on classification, labelling and packaging (CLP) of substances and mixtures. Ver 5.0. Helsinki, Finland.

[etc4824-bib-0023] European Chemicals Agency . 2017b. Read‐across assessment framework (RAAF). Helsinki, Finland.

[etc4824-bib-0024] European Chemicals Agency . 2017c. Guidance on information requirements and chemical safety assessment. Chapter R.7b: Endpoint specific guidance. Ver 4.0. Helsinki, Finland.

[etc4824-bib-0025] European Chemicals Agency . 2017d. Report on the current status of regulatory applicability of non‐animal approaches under the REACH, CLP and biocidal products regulations. Helsinki, Finland.

[etc4824-bib-0026] European Chemicals Agency . 2018. Guidance on the biocidal products regulation, Vol 4: Environment, Part A: Information Requirements. ECHA‐18‐G‐06‐EN. Helsinki, Finland.

[etc4824-bib-0027] European Commission . 1991 Council Directive of 15 July 1991 concerning the placing of plant protection products on the market (91/414/EEC). Official J Eur Union L230:1.

[etc4824-bib-0028] European Commission . 2006 Regulation (EC) No 1907/2006 of the European Parliament and of the Council of 18 December 2006 concerning the Registration, Evaluation, Authorisation and Restriction of Chemicals (REACH), establishing a European Chemicals Agency, amending Directive 1999/4. Official J Eur Union L396:374–375.

[etc4824-bib-0029] European Commission . 2009a Regulation (EC) No 1107/2009 of the European Parliament and of the Council of 21 October 2009 concerning the placing of plant protection products on the market and repealing Council Directives 79/117/EEC and 91/414/EEC. Official J Eur Union L309:1–50.

[etc4824-bib-0030] European Commission . 2009b Regulation (EC) No 1223/2009 of the European Parliament and of the Council of 30 November 2009 on cosmetic products (text with EEA relevance). Official J Eur Union L342:59–209.

[etc4824-bib-0031] European Commission . 2010 Directive 2010/63/EU of the European Parliament and of the Council of 22 September 2010 on the protection of animals used for scientific purposes. Official J Eur Union L276:34–79.

[etc4824-bib-0032] European Commission . 2013 Commission Regulation (EU) No 283/2013 of 1 March 2013 setting out the data requirements for active substances, in accordance with Regulation (EC) No 1107/2009 of the European Parliament and of the Council concerning the placing of plant protection products. Official J Eur Union L93:1–84.

[etc4824-bib-0033] European Commission . 2014. EURL ECVAM recommendation on the zebrafish embryo acute toxicity test method (ZFET) for acute aquatic toxicity testing. Ispra, Italy.

[etc4824-bib-0034] European Food Safety Authority . 2005 Opinion of the Scientific Panel on Animal Health and Welfare (AHAW) on a request from the commission related to the aspects of the biology and welfare of animals used for experimental and other scientific purposes. EFSA J 3:292.

[etc4824-bib-0035] European Food Safety Authority . 2006 Opinion of the Scientific Panel on Plant Health, Plant Protection Products and their Residues (PPR) on a request from EFSA related to the assessment of the acute and chronic risk to aquatic organisms with regard to the possibility of lowering the uncertainty factor if additional species were tested. EFSA J 4:301.

[etc4824-bib-0036] European Food Safety Authority . 2013 Guidance on tiered risk assessment for plant protection products for aquatic organisms in edge‐of‐field surface waters. EFSA J 11:3290.

[etc4824-bib-0037] European Medicines Agency . 2006. Guideline on the environmental risk assessment of medicinal products for human use. Ref EMEA/CHMP/SWP/4447/00 corr 2. London, UK.

[etc4824-bib-0038] European Medicines Agency . 2016. ICH guideline S1 regulatory notice on changes to core guideline on rodent carcinogenicity. EMA/CHMP/ICH/536328/2013 Rev. 1. London, UK.

[etc4824-bib-0039] Fischer M , Belanger SE , Berckmans P , Bernhard MJ , Bláha L , Coman Schmid DE , Dyer SD , Haupt T , Hermens JLM , Hultman MT , Laue H , Lillicrap A , Mlnaříková M , Natsch A , Novák J , Sinnige TL , Tollefsen KE , von Niederhäusern V , Witters H , Županič A , Schirmer K . 2019 Repeatability and reproducibility of the RTgill‐W1 cell line assay for predicting fish acute toxicity. Toxicol Sci 169:353–364. 30825313 10.1093/toxsci/kfz057 PMC6542334

[etc4824-bib-0040] German Advisory Council on the Environment . 2007. Pharmaceuticals in the environment. Statement April 2007. No. 12. Berlin, Germany.

[etc4824-bib-0041] Green J , Wheeler JR . 2013 The use of carrier solvents in regulatory aquatic toxicology testing: Practical, statistical and regulatory considerations. Aquat Toxicol 144–145:242–249. 10.1016/j.aquatox.2013.10.004 24185102

[etc4824-bib-0042] Groh KJ , Tollefsen KE . 2015 The challenge: Adverse outcome pathways in research and regulation—Current status and future perspectives. Environ Toxicol Chem 34:1935–1937. 10.1002/etc.3042 26313029

[etc4824-bib-0043] Gunnarsson L , Snape JR , Verbruggen B , Owen SF , Kristiansson E , Margiotta‐Casaluci L , Österlund T , Hutchinson K , Leverett D , Marks B , Tyler CR . 2019 Pharmacology beyond the patient—The environmental risks of human drugs. Environ Int 129:320–332. 31150974 10.1016/j.envint.2019.04.075

[etc4824-bib-0045] HUGIN SWiFT . n.d. Welcome to the HUGIN SWiFT project site. Norwegian Institute for Water Research, Oslo, Norway. [cited 2020 June 18]. Available from: http://swift.hugin.com/

[etc4824-bib-0044] Hunn JB . 1989 *History of Acute Toxicity Tests with Fish, 1863–1987*, Vol 98—Investigations in Fish Control. US Fish and Wildlife Service, Springfield VA.

[etc4824-bib-0046] Hutchinson TH , Barrett S , Buzby M , Constable D , Hartmann A , Hayes E , Huggett D , Laenge R , Lillicrap AD , Straub JO , Thompson RS . 2003 A strategy to reduce the numbers of fish used in acute ecotoxicity testing of pharmaceuticals. Environ Toxicol Chem 22:3031–3036. 14713046 10.1897/02-558

[etc4824-bib-0047] Hutchinson TH , Scholz N , Guhl W . 1998 Analysis of the ECETOC aquatic toxicity (EAT) database IV—Comparative toxicity of chemical substances to freshwater versus saltwater organisms. Chemosphere 36:143–153.

[etc4824-bib-0048] Hutchinson TH , Wheeler JR , Gourmelon A , Burden N . 2016 Promoting the 3Rs to enhance the OECD fish toxicity testing framework. Regul Toxicol Pharmacol 76:231–233. 26873775 10.1016/j.yrtph.2016.02.006

[etc4824-bib-0049] International Organization for Standardization . 2007 Water quality—Determination of the acute toxicity of wastewater to zebrafish eggs (*Danio rerio*). ISO 15088:2007(E). Geneva, Switzerland.

[etc4824-bib-0050] International Organization for Standardization . 2019. Water quality—Determination of acute toxicity of water samples and chemicals to a fish gill cell line (RTgill‐W1). ISO 21115:2019. Geneva, Switzerland.

[etc4824-bib-0051] Jeram S , Sintes JMR , Halder M , Fentanes JB , Sokull‐Klüttgen B , Hutchinson TH . 2005 A strategy to reduce the use of fish in acute ecotoxicity testing of new chemical substances notified in the European Union. Regul Toxicol Pharmacol 42:218–224. 15949882 10.1016/j.yrtph.2005.04.005

[etc4824-bib-0052] Kleinow KM , Melancon MJ , Lech JJ . 1987 Biotransformation and induction: Implications for toxicity, bioaccumulation and monitoring of environmental xenobiotics in fish. Environ Health Perspect 71:105–119. 3297653 10.1289/ehp.8771105 PMC1474347

[etc4824-bib-0053] Knöbel M , Busser FJM , Rico‐Rico Á , Kramer NI , Hermens JLM , Hafner C , Tanneberger K , Schirmer K , Scholz S . 2012 Predicting adult fish acute lethality with the zebrafish embryo: Relevance of test duration, endpoints, compound properties, and exposure concentration analysis. Environ Sci Technol 46:9690–9700. 22835061 10.1021/es301729q

[etc4824-bib-0054] LaLone CA , Villeneuve DL , Lyons D , Helgen HW , Robinson SL , Swintek JA , Saari TW , Ankley GT . 2016 Editor's highlight: Sequence Alignment to Predict Across Species Susceptibility (SeqAPASS): A web‐based tool for addressing the challenges of cross‐species extrapolation of chemical toxicity. Toxicol Sci 153:228–245. 27370413 10.1093/toxsci/kfw119

[etc4824-bib-0055] Lammer E , Carr GJ , Wendler K , Rawlings JM , Belanger SE , Braunbeck T . 2009 Is the fish embryo toxicity test (FET) with the zebrafish (*Danio rerio*) a potential alternative for the fish acute toxicity test? Comp Biochem Physiol C Toxicol Pharmacol 149:196–209. 19095081 10.1016/j.cbpc.2008.11.006

[etc4824-bib-0056] Le Page G , Gunnarsson L , Snape J , Tyler CR . 2017 Integrating human and environmental health in antibiotic risk assessment: A critical analysis of protection goals, species sensitivity and antimicrobial resistance. Environ Int 109:155–169. 28964562 10.1016/j.envint.2017.09.013

[etc4824-bib-0057] Leung KMY , Morritt D , Wheeler JR , Whitehouse P , Sorokin N , Toy R , Holt M , Crane M . 2001 Can saltwater toxicity be predicted from freshwater data? Mar Pollut Bull 42:1007–1013. 11763210 10.1016/s0025-326x(01)00135-7

[etc4824-bib-0058] Lillicrap A , Belanger S , Burden N , Du Pasquier D , Embry MR , Halder M , Lampi MA , Lee L , Norberg‐King T , Rattner BA , Schirmer K , Thomas P . 2016 Alternative approaches to vertebrate ecotoxicity tests in the 21st century: A review of developments over the last 2 decades and current status. Environ Toxicol Chem 35:2637–2646. 27779828 10.1002/etc.3603

[etc4824-bib-0059] Lillicrap A , Moe SJ , Wolf R , Connors KA , Rawlings JM , Landis WG , Madsen A , Belanger SE . 2020 Evaluation of a Bayesian network for predicting acute fish toxicity from fish embryo toxicity data. Integr Environ Assess Manag 16:452–460. 32125082 10.1002/ieam.4258

[etc4824-bib-0060] Luckert C , Schulz C , Lehmann N , Thomas M , Hofmann U , Hammad S , Hengstler JG , Braeuning A , Lampen A , Hessel S . 2017 Comparative analysis of 3D culture methods on human HepG2 cells. Arch Toxicol 91:393–406. 26872951 10.1007/s00204-016-1677-z

[etc4824-bib-0061] Maltby L , Blake N , Brock TCM , Van den Brink PJ . 2005 Insecticide species sensitivity distributions: Importance of test species selection and relevance to aquatic ecosystems. Environ Toxicol Chem 24:379–388. 15719998 10.1897/04-025r.1

[etc4824-bib-0062] Martin OV , Adams J , Beasley A , Belanger S , Breton RL , Brock TCM , Buonsante VA , Galay Burgos M , Green J , Guiney PD , Hall T , Hanson M , Harris MJ , Henry TR , Huggett D , Junghans M , Laskowski R , Maack G , Moermond CTA , Panter G , Pease A , Poulsen V , Roberts M , Rudén C , Schlekat CE , Schoeters I , Solomon KR , Staveley J , Stubblefield B , Sumpter JP , Warne MSJ , Wentsel R , Wheeler JR , Wolff BA , Yamazaki K , Zahner H , Ågerstrand M . 2019 Improving environmental risk assessments of chemicals: Steps towards evidence‐based ecotoxicology. Environ Int 128:210–217. 31059916 10.1016/j.envint.2019.04.053

[etc4824-bib-0063] Mayer P , Reichenberg F . 2006 Can highly hydrophobic organic substances cause aquatic baseline toxicity and can they contribute to mixture toxicity? Environ Toxicol Chem 25:2639–2644. 17022404 10.1897/06-142r.1

[etc4824-bib-0064] Maynard SK , Clook M , Benstead R , Handley J , Hutchinson TH , Ryder K , Sheahan D , Snape JR , Whale G , Wheeler JR , Burden N . 2017 A cross sector review of global requirements for acute fish toxicity testing—Opportunities for harmonisation and implementation of the 3Rs. *Abstracts*, 27th Annual Meeting, Society of Environmental Toxicology and Chemistry Europe, Brussels, Belgium, May 7–11, poster no. TUPC22.

[etc4824-bib-0065] Moermond CTA , Kase R , Korkaric M , Ågerstrand M . 2016 CRED: Criteria for reporting and evaluating ecotoxicity data. Environ Toxicol Chem 35:1297–1309. 26399705 10.1002/etc.3259

[etc4824-bib-0066] National Centre for the Replacement, Refinement and Reduction of Animals in Research . n.d. London, UK. [cited 2020 June 18]. Available from: www.nc3rs.org.uk/the-3rs

[etc4824-bib-0067] National Centre for the Replacement, Refinement and Reduction of Animals in Research . 2018 Innovation platform: Exploiting 3Rs technologies. Maximise. London, UK. [cited 2020 June 18]. Available from: https://www.nc3rs.org.uk/crackit/maximise

[etc4824-bib-0068] National Research Council . 2012 Exposure Science in the 21st Century. National Academies Press, Washington, DC. 24901193

[etc4824-bib-0069] National Toxicology Program . 2020 Fish acute toxicity. National Institute of Environmental Health Sciences, Research Triangle Park, NC, USA. [cited 2020 June 16]. Available from: https://ntp.niehs.nih.gov/whatwestudy/niceatm/test-method-evaluations/acute-systemic-tox/fish/index.html

[etc4824-bib-0070] Natsch A , Laue H , Haupt T , von Niederhäusern V , Sanders G . 2018 Accurate prediction of acute fish toxicity of fragrance chemicals with the RTgill‐W1 cell assay. Environ Toxicol Chem 37:931–941. 29105821 10.1002/etc.4027

[etc4824-bib-0071] Nichols J , Erhardt S , Dyer S , James M , Moore M , Plotzke K , Segner H , Schultz I , Thomas K , Vasiluk L , Weisbrod A . 2007 Use of in vitro absorption, distribution, metabolism, and excretion (ADME) data in bioaccumulation assessments for fish. Hum Ecol Risk Assess 13:1164–1191.

[etc4824-bib-0072] Organisation for Economic Co‐operation and Development . 1987. Test No. 401: Acute Oral Toxicity. Paris: OECD (OECD Guidelines for the Testing of Chemicals, Section 4). OECD Publishing, Paris.

[etc4824-bib-0108] Organisation for Economic Co‐operation and Development . 2000. Guidance document on the recognition, assessment, and use of clinical signs as humane endpoints for experimental animals used in safety evaluation. Series on Testing and Assessment, No. 19. ENV/JM/MONO(2000)7. Paris, France.

[etc4824-bib-0073] Organisation for Economic Co‐operation and Development . 2002 Test No. 420: Acute oral toxicity—Fixed dose procedure. *OECD Guidelines for the Testing of Chemicals*. Paris, France.

[etc4824-bib-0074] Organisation for Economic Co‐operation and Development . 2011a. Validation report (phase 1) for the zebrafish embryo toxicity test, part I. Series on Testing and Assessment, No. 157. ENV/JM/MONO(2011)37. Paris, France.

[etc4824-bib-0075] Organisation for Economic Co‐operation and Development . 2011b. Validation report (phase 1) for the zebrafish embryo toxicity test, part 2. Series on Testing and Assessment, No. 157. ENV/JM/MONO(2011)40. Paris, France.

[etc4824-bib-0076] Organisation for Economic Co‐operation and Development . 2012. Report of the test method validation zebrafish embryo toxicity test (ZFET) phase 2—Testing of 13 chemicals. Series on Testing and Assessment, No. 179. ENV/JM/MONO(2012)25. Paris, France.

[etc4824-bib-0077] Organisation for Economic Co‐operation and Development . 2013 Test No. 236: Fish embryo acute toxicity (FET) test. *OECD Guidelines for the Testing of Chemicals*. Paris, France.

[etc4824-bib-0078] Organisation for Economic Co‐operation and Development . 2014. Fish toxicity testing framework. Series on Testing and Assessment, No. 171. ENV/JM/MONO(2012)16. Paris, France.

[etc4824-bib-0079] Organisation for Economic Co‐operation and Development . 2015 Test No. 240: Medaka extended one generation reproduction test (MEOGRT). *OECD Guidelines for the Testing of Chemicals*. Paris, France.

[etc4824-bib-0080] Organisation for Economic Co‐operation and Development . 2016a Test No. 223: Avian acute oral toxicity test. *OECD Guidelines for the Testing of Chemicals*. Paris, France.

[etc4824-bib-0081] Organisation for Economic Co‐operation and Development . 2016b. Guidance document on the reporting of defined approaches to be used within integrated approaches to testing and assessment. Series on Testing and Assessment, No. 255. ENV/JM/MONO(2016)28. Paris, France.

[etc4824-bib-0082] Organisation for Economic Co‐operation and Development . 2017a. Guidance on grouping of chemicals, second edition. Series on Testing and Assessment, No. 194. ENV/JM/MONO(2014)4. Paris, France.

[etc4824-bib-0083] Organisation for Economic Co‐operation and Development . 2017b Test No. 402: Acute dermal toxicity. *OECD Guidelines for the Testing of Chemicals*. Paris, France.

[etc4824-bib-0084] Organisation for Economic Co‐operation and Development . 2018 Test No. 433: Acute inhalation toxicity: Fixed concentration procedure. *OECD Guidelines for the Testing of Chemicals*. Paris, France.

[etc4824-bib-0085] Organisation for Economic Co‐operation and Development . 2019a Test No. 203: Fish, acute toxicity test. *OECD Guidelines for the Testing of Chemicals*. Paris, France.

[etc4824-bib-0086] Organisation for Economic Co‐operation and Development . 2019b. Guiding principles and key elements for establishing a weight of evidence for chemical assessment. Series on Testing and Assessment, No. 311. ENV/JM/MONO(2019)31. Paris, France.

[etc4824-bib-0087] Organisation for Economic Co‐operation and Development . 2019c Saving costs in chemicals management: How the OECD ensures benefits to Society. Paris, France. [cited 2020 June 16]. Available from: https://www.oecd.org/chemicalsafety/saving-costs-in-chemicals-management-9789264311718-en.htm

[etc4824-bib-0088] Organisation for Economic Co‐operation and Development . 2019d. Guidance document on aquatic toxicity testing of difficult substances and mixtures. Series on Testing and Assessment, No. 23. ENV/JM/MONO(2000)6/REV1. Paris, France.

[etc4824-bib-0089] Prior H , Casey W , Kimber I , Whelan M , Sewell F . 2019 Reflections on the progress towards non‐animal methods for acute toxicity testing of chemicals. Regul Toxicol Pharmacol 102:30–33. 30578838 10.1016/j.yrtph.2018.12.008

[etc4824-bib-0090] Rawlings JM , Belanger SE , Connors KA , Carr GJ . 2019 Fish embryo tests and acute fish toxicity tests are interchangeable in the application of the threshold approach. Environ Toxicol Chem 38:671–681. 30615221 10.1002/etc.4351

[etc4824-bib-0091] Redman AD , Parkerton TF , Leon Paumen M , Butler JD , Letinski DJ , den Haan K . 2017 A re‐evaluation of PETROTOX for predicting acute and chronic toxicity of petroleum substances. Environ Toxicol Chem 36:2245–2252. 28106281 10.1002/etc.3744

[etc4824-bib-0092] Rufli H , Springer TA . 2011 Can we reduce the number of fish in the OECD acute toxicity test? Environ Toxicol Chem 30:1006–1011. 21309020 10.1002/etc.465

[etc4824-bib-0093] Schmuck R , Pfluger W , Grau R , Hollihn U , Fischer R . 1994 Comparison of short‐term aquatic toxicity: Formulation vs active ingredients of pesticides. Arch Environ Contam Toxicol 26:240–250.

[etc4824-bib-0094] Sewell F , Aggarwal M , Bachler G , Broadmeadow A , Gellatly N , Moore E , Robinson S , Rooseboom M , Stevens A , Terry C , Burden N . 2017a The current status of exposure‐driven approaches for chemical safety assessment: A cross‐sector perspective. Toxicology 389:109–117. 28774667 10.1016/j.tox.2017.07.018

[etc4824-bib-0095] Sewell F , Doe J , Gellatly N , Ragan I , Burden N . 2017b Steps towards the international regulatory acceptance of non‐animal methodology in safety assessment. Regul Toxicol Pharmacol 89:50–56. 28689746 10.1016/j.yrtph.2017.07.001

[etc4824-bib-0096] Sewell F , Ragan I , Indans I , Marczylo T , Stallard N , Griffiths D , Holmes T , Smith P , Horgan G . 2018 An evaluation of the fixed concentration procedure for assessment of acute inhalation toxicity. Regul Toxicol Pharmacol 94:22–32. 29309809 10.1016/j.yrtph.2018.01.001

[etc4824-bib-0097] Sewell F , Ragan I , Marczylo T , Anderson B , Braun A , Casey W , Dennison N , Griffiths D , Guest R , Holmes T , van Huygevoort T , Indans I , Kenny T , Kojima H , Lee K , Prieto P , Smith P , Smedley J , Stokes WS , Wnorowski G , Horgan G . 2015 A global initiative to refine acute inhalation studies through the use of “evident toxicity” as an endpoint: Towards adoption of the fixed concentration procedure. Regul Toxicol Pharmacol 73:770–779. 26505531 10.1016/j.yrtph.2015.10.018

[etc4824-bib-0098] Tanneberger K , Knöbel M , Busser FJM , Sinnige TL , Hermens JLM , Schirmer K . 2013 Predicting fish acute toxicity using a fish gill cell line–based toxicity assay. Environ Sci Technol 47:1110–1119. 23227966 10.1021/es303505z

[etc4824-bib-0099] Thomas P , Dawick J , Lampi M , Lemaire P , Presow S , van Egmond R , Arnot JA , Mackay D , Mayer P , Galay Burgos M . 2015 Application of the activity framework for assessing aquatic ecotoxicology data for organic chemicals. Environ Sci Technol 49:12289–12296. 26378470 10.1021/acs.est.5b02873

[etc4824-bib-0100] US Environmental Protection Agency . 2012. Avian acute oral toxicity test. OCSPP 850.2100. Washington DC.

[etc4824-bib-0101] US Environmental Protection Agency . 2016a. Freshwater and saltwater fish acute toxicity test. OCSPP 850.1075. Washington DC.

[etc4824-bib-0102] US Environmental Protection Agency . 2016b Interspecies correlation estimation. Washington, DC. [cited 2020 June 18]. Available from: www3.epa.gov/webice/

[etc4824-bib-0103] US Environmental Protection Agency . 2017 USEPA OPP initiative to modernize the acute “6‐pack”—Update to the PPDC. Washington, DC. [cited 2020 June 18]. Available from: www.epa.gov/sites/production/files/2017-04/documents/session-3-2st-century-toxicology-update.pdf

[etc4824-bib-0104] US Environmental Protection Agency . 2019 Administrator memo prioritizing efforts to reduce animal testing, September 10, 2019. Washington, DC. [cited 2020 June 18]. Available from: https://www.epa.gov/research/administrator-memo-prioritizing-efforts-reduce-animal-testing-september-10-2019

[etc4824-bib-0105] Weyers A , Sokull‐Klüttgen B , Baraibar‐Fentanes J , Vollmer G . 2000 Acute toxicity data: A comprehensive comparison of results of fish, daphnia, and algae tests with new substances notified in the European Union. Environ Toxicol Chem 19:1931–1933.

[etc4824-bib-0106] Wheeler J , Grist EP , Leung KM , Morritt D , Crane M . 2002 Species sensitivity distributions: Data and model choice. Mar Pollut Bull 45:192–202. 12398385 10.1016/s0025-326x(01)00327-7

[etc4824-bib-0107] Wheeler JR , Maynard SK , Crane M . 2014 Are acute and chronic saltwater fish studies required for plant protection and biocidal product active substance risk assessment? Environ Toxicol Chem 33:703–707. 24288251 10.1002/etc.2478

