# Supplementary material for: Key Opportunities to Replace, Reduce, and Refine Regulatory Fish Acute Toxicity Tests
Source: Environ Toxicol Chem. 2020 Aug 24;39(10):2076–89. doi: 10.1002/etc.4824 (PMC7754335; doi:10.1002/etc.4824)

**Supporting information 1.**

An example tiered approach to testing so that adequate information is available to assess the potential environmental fate and effects of pharmaceuticals while minimising the cost to industry. This demonstrates the triggers for fish acute toxicity tests of pharmaceuticals for USA registrations. Figure taken from FDA (1998). ‘Guidance for Industry, Environmental Assessment of Human Drug and Biologics Applications’ CDER, CBER, FDA July 1998. [www.fda.gov/media/70809/download](http://www.fda.gov/media/70809/download). Last accessed on 16 June 2020.


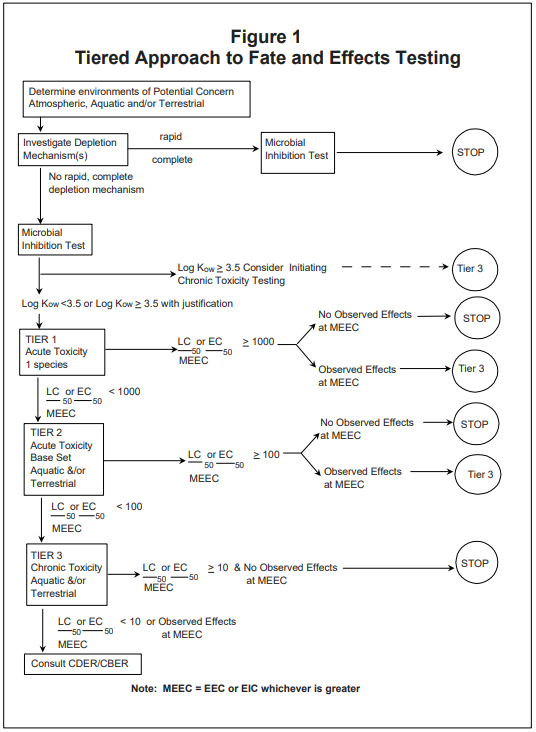

Supplement: Supplementary file 1 — Supporting information. [file ETC-39-2076-s001.docx]
