# Supplementary material for: Key Opportunities to Replace, Reduce, and Refine Regulatory Fish Acute Toxicity Tests
Source: Environ Toxicol Chem. 2020 Aug 24;39(10):2076–89. doi: 10.1002/etc.4824 (PMC7754335; doi:10.1002/etc.4824)
Supplement: Supplementary file 2 — Supporting information. [file ETC-39-2076-s002.docx]

Supporting Information 2. Case studies using alternative approaches to waive *in vivo* fish acute toxicity tests under European Union Regulation (EC) No 1907/2006 Registration, Evaluation, Authorisation and Restriction of Chemicals (REACH).

| **CASE STUDY 1 – use of QSAR endpoint, supporting analogue data, experimental considerations**  Substance: 1,3-butadiene CAS: 106-99-0 Context: European Union Council Regulation (EEC) No. 793/93 on the evaluation and control of the risks of existing substances  Key physico-chemical properties^1^:   - water solubility: 735 mg/L at 20 oC - logKow: 1.99 - Henry’s Law Constant (HLC): 7150 Pa m3/mol at 20 ^o^C (considered likely to  partition from the water phase to the atmosphere)   Experimental aquatic toxicity data are not available. Due to the substances physico-chemical properties aquatic ecotoxicity testing may not be feasible.  Assessment under European Council Regulation 793/931 on the evaluation and control of the risks of “existing” substances^2^ considered two approaches for the acute toxicity to fish endpoint. A QSAR based on the logKow and non-polar narcosis predicted a 96-h LC_50_ of 45 mg/L for Fathead minnow. Read across to two structurally similar substances listed the following experimental and QSAR endpoints:  2-methyl-1,3-butadience (CAS: 78-79-5):   - experimental 96-h LC_50_ 74.8 mg/L for Fathead minnow - experimental 96-h LC_50_ 42.5 mg/L for Bluegill sunfish - QSAR 96-h LC_50_ 30.8 mg/L   3-pentadiene (CAS: 504-60-9):   - experimental 96-h LC_50_ 140 mg/L for Fathead minnow - QSAR 96-h LC50 30.8 mg/L   Analogue experimental endpoints were considered in general agreement (within a factor of 10) with predicted endpoints adding confidence to the QSAR prediction for 1,3-butadiene. In addition, it was noted that QSARs endpoints were generally lower than experimental endpoints.  The predicted endpoints for 1,3-butadiene were used with associated assessment factors to derive an aquatic Predicted No Effect Concentration (PNEC). The PNEC was used to assess risk to the aquatic environment which indicated low risk. On this basis, the assessment considered an acute toxicity to fish study was not required.  This example has since been cited in ECHA’s Guidance on information requirements and chemical safety assessment, Chapter R.6: QSARs and grouping of chemicals^3^.  ^1^ Taken from EU REACH Registration ECHA database available at: <https://echa.europa.eu/registration-dossier/-/registered-dossier/15570/1> accessed 2019-07-10  ^2^ European Chemicals Bureau (ECB) (2002) European Union Risk Assessment Report for 1,3-butadiene CAS 106-99-0. Available at: <https://echa.europa.eu/information-on-chemicals/information-from-existing-substances-regulation/-/substance-rev/2448/term>  ^3^ European Chemical Agency (2008) Guidance on information requirements and chemical safety assessment, Chapter R.6: QSARs and grouping of chemicals. Available at: <https://echa.europa.eu/documents/10162/13632/information_requirements_r6_en.pdf/77f49f81-b76d-40ab-8513-4f3a533b6ac9> |
| --- |
| **CASE STUDY 2 – use of category approach with supporting analogue data, experimental considerations**  Substance: 1-tetradecene, polymer with 1-dodecene, distn. residues, hydrogenated, C36-84 fraction CAS: 883233-91-8 Context: European Union Regulation (EC) No 1907/2006 Registration, Evaluation, Authorisation and Restriction of Chemicals (REACH) [Compliance Check]  Key physico-chemical properties^1^:  - water solubility: <0.1 mg/L (based on analogue read-across) - logKow: >6.5 (based on analogue read-across)  The substance is part of a category for poly alpha olefins (POA). Eight acute toxicity to fish studies are available for the category using Rainbow trout and Sheepshead minnow. The POA substances are considered to act via non-polar narcosis with the low water solubility limiting exposure for acute aquatic toxicity. Due to the low water solubility across the category, the endpoints are based on Water Accommodated Fractions. No effects were observed at the highest WAF loading of 1000 mg/L. On this basis, the LL_50_ for the substance is considered >1000 mg/L and an acute toxicity to fish study has not been performed with the endpoint read-across the category.  ^1^ Taken from EU REACH Registration ECHA database available at: <https://echa.europa.eu/registration-dossier/-/registered-dossier/12597/1> accessed 2019-07-11 |
| **CASE STUDY 3 – use of FET, weight of evidence, experimental considerations**  Substance: Bis(piperidinothiocarbonyl)hexasulphide CAS: 971-15-3  Context: European Union Regulation (EC) No 1907/2006 Registration, Evaluation, Authorisation and Restriction of Chemicals (REACH) [Dossier Evaluation]  Key physico-chemical properties^1^:  - water solubility: 0.01 mg/L at 20 ^o^C  - logKow: 4.43 (QSAR) and 6.2 (experimental) at 20‌ ^o^C  The ecotoxicity data package includes a FET (OECD 236) study (considered Reliability 1) using Zebrafish in which no mortality was observed up to quoted limit of water solubility. A 33-d FELS (OECD 210) is also available where no adverse effects were observed up to the quoted limit of solubility. The data package was considered under a Dossier Evaluation and discussed by experts – further acute toxicity to fish data were not required^2^.  ^1^ Taken from EU REACH Registration ECHA database available at: <https://echa.europa.eu/registration-dossier/-/registered-dossier/5749/1> accessed 2019-07-11  ^2^ Information available at: <https://echa.europa.eu/pact?p_p_id=disspact_WAR_disspactportlet&p_p_lifecycle=0&_disspact_WAR_disspactportlet_substanceId=100.012.307&_disspact_WAR_disspactportlet_jspPage=%2Fhtml%2Fportlet%2Fdisspact%2FdetailsPage%2Fview_detailsPage.jsp> accessed 2019-07-11 |
| **CASE STUDY 4 – use of weight of evidence, analogues, exposure considerations**  Substance: Imidazole CAS: 288-32-4 Context: European Union Regulation (EC) No 1907/2006 Registration, Evaluation, Authorisation and Restriction of Chemicals (REACH) [Substance Evaluation]  Key physico-chemical properties^1^:  - water solubility: 663000 mg/L at 20 oC  - logKow: -0.02 at 20 oC  - considered readily biodegradable according to OECD 301  Ecotoxicity data were considered under Substance Evaluation^2^. A 48-h LC_50_ of 284 mg/L for a non-standard OECD 203 test species was included. The study was not run to GLP, full study details were not available and analytical verification was not included. Acute toxicity to fish 96-LC_50_ endpoints for two analogues (1-methylimidazole CAS: 916-47-4 and 2-methylimidazole CAS 693-98-1) were provided as supporting information. Additional data and QSAR analysis suggested fish were not the most sensitive trophic level and available algal data were sufficient to conduct the environmental risk assessment. Overall, the assessment considered a weight of evidence that the substance was of low acute toxicity to fish and further acute toxicity to fish data were not required.  ^1^ Taken from EU REACH Registration ECHA database available at: accessed 2019-07-11  ^2^ Details available at: <https://echa.europa.eu/information-on-chemicals/evaluation/community-rolling-action-plan/corap-table/-/dislist/details/0b0236e1807e3ee1> accessed 2019-07-11 |
