# Supplementary material for: Key Opportunities to Replace, Reduce, and Refine Regulatory Fish Acute Toxicity Tests
Source: Environ Toxicol Chem. 2020 Aug 24;39(10):2076–89. doi: 10.1002/etc.4824 (PMC7754335; doi:10.1002/etc.4824)
Supplement: Supplementary file 3 — Supporting information. [file ETC-39-2076-s003.docx]

Supporting information 3. Comparison of notable differences between commonly used fish acute toxicity test guidelines. DO = dissolved oxygen.

| **Parameter** | **US: OCSPP 850.1075** | **OECD 203 (Updated 2019)** | **Japan: JMAFF** |
| --- | --- | --- | --- |
| Test species permitted/recommended: | *Oncorhynchus mykiss* - Rainbow trout and, *Lepomis macrochirus* - Bluegill sunfish, are the preferred species; also *Menidia sp.* (silversides) preferred for saltwater testing.  All other species included: *Salmo salar -* Atlantic salmon, *Salvelinus fontinalis -* Brook trout, *Ictalurus punctatus -* Channel catfish*;* *Oncorhynchus kisutch -* Coho salmon, *Cyprinus carpio - C*ommon carp, *Pimephales promelas -* fathead minnow, *Poecilia reticulata –* Guppy, *Oryzias latipes –* Medaka, and *Danio rerio* - Zebrafish. Saltwater: *Menidia menidia -* Atlantic silverside, *Menidia beryllina* - inland silverside, *Menidia peninsula -* tidewater silverside, and *Cyprinodon variegatus* - Sheepshead minnow | *Pimephales promelas -* Fathead minnow, *Oncorhynchus mykiss -* Rainbow trout*, Danio rerio - Z*ebrafish, *Cyprinus carpio* - Carp, *Oryzias latipes -* Japanese Medaka, *Poecilia reticulata -* Guppy, *Lepomis macrochirus –* Bluegill sunfish, *Gasterosteus aculeatus -* Three-spined stickleback, *Cyprinodon variegatus -* Sheepshead minnow, *Dicentrarchus labrax -* European sea bass, *Pagrus major* - Sea bream | *Pimephales promelas -* Fathead minnow*, Oncorhynchus mykiss -* Rainbow trout*, Danio rerio -* Zebrafish, *Cyprinus carpio* - Carp, *Oryzias latipes -* Japanese Medaka, *Poecilia reticulata -* Guppy, *Lepomis macrochirus –* Bluegill sunfish |
| Age/size of test organisms | Juvenile fish <3.0 grams | Juvenile, same age, similar size - length is recommended according to each species. Growth (max. 8cm allowed for European seabass) | 2.0 ± 1.0cm for most fish, 4.0 ± 2.0cm for carp, 2.3 ± 1.2cm for J.Medaka, 5.0 ± 1.0cm for trout. Use juvenile fish. Fish used should be the same age and be of normal size and appearance for their age |
| Pre-exposure acclimation period | Minimum of 12 days if acquired by another lab/source, holding period should be minimum 14 days, minimum 7 days for acclimation to test conditions | Minimum 9 days if acquired by another lab/source, at least 7 days to test conditions | 12 days if acquired by another lab, at least 9 days acclimation to the test conditions before the study |
| Acclimatisation mortality rate acceptability | (A) Mortalities of greater than 10% of the population in the 7 days of acclimation: rejection of entire batch; (B) Mortalities of between 5 and 10% of the population during the 7 days of acclimation: acclimation continued for additional 7 days; (C) Mortalities of less than 5% of the population during the 7 days of acclimation: acceptance of batch. | (A) mortalities >10% of population: rejection of entire batch or acclimatisation should continue for 7 additional days; (B) mortalities between 5 and 10% of population: acclimatisation should continue for 7 additional days; (C) mortalities of <5% of population: acceptance of batch. | (A) If during the 7-day period following the stabilization period (the 2 days following the commencement of acclimatization) the mortality rate exceeds 10% of the individuals in a group, that group should be excluded (B) If the group mortality rate is 5-10%, and is still ≥5% after acclimatization for 7 days, exclude the group, or continue acclimatization until the mortality rate falls below 5% (C) If the group mortality rate is less than 5%, the fish in that group may be used in the studies |
| Dilution water | Clean, surface, well, or reconstituted water  Total alkalinity > 20 mg/L (as CaCO3)  Unionized Ammonia ≤ 1 μg/L  Residual chlorine < 10 μg/L | For fresh water: clean surface water, ground water or reconstituted water, dechlorinated drinking water may also be used  For marine: reconstituted water is preferred to seawater and can be prepared by adding commercial sea salts | Use dechlorinated tap water, natural water supplies, or reconstituted water. Do not use water that contains hazardous substances for the study. Use water after its quality has been demonstrated to be favourable to the survival and development of fish, from the same source as the water in which they were bred. |
| Test chamber size | The size, shape, and depth of the test vessel should be appropriate if the specified flow rate and loading requirements can be achieved. Should be large enough for the DO not to fall below recommended levels, to comply with the loading criteria | Large enough to keep fish free of stress and comply with the loading criteria, also randomly positioned at test area shielded from unwanted disturbance | n/a |
| Test volume | Each test vessel should contain an equal volume of test solution and an equal number of fish. Test vessels should be loosely covered to reduce the loss of test solution or dilution water due to evaporation, to minimize the entry of dust or other particulates into solutions, and to prevent loss of test fish | Dependent on size of vessels | n/a |
| Loading | Static or static-renewal tests: ≤0.8 g wet weight per litre; Flow-through test: ≤0.5 g/L per 24 hours and <5 g/L at any time | Static or static-renewal tests: ≤0.8 g wet weight per litre; Flow-through test: ≤0.5 g/L per 24 hours and <5 g/L at any time | For static and semi-static, at least 1 L of test solution per gram of test fish body weight. A still higher population may be used in flow-through studies |
| Flow rate | 2.7 L / hr | For flow-through systems, the recommended maximum loading is 0.5 g wet weight fish/L per 24 hours (example: in a 10 L tank with a flow rate of 5 tank volumes per 24 hours, a total of 50 L pass through the tank in 24 hours. With 25 g fish, this corresponds to 25 g in 50 L in 24 hours equivalent to 0.5 g/L in 24 hours) | n/a |
| Geometric factor | n/a | 1.6-1.8 preferable, should not exceed 2.2 | >2.2 |
| # Replicates per concentration | 1 minimum, 2 preferred - Replicates should be physically separated since the test vessel is the experimental unit - should be randomised in the test area | No replication required | n/a |
| Weight of each fish | Juvenile fish <3.0 grams | Initial fish weight should be measured in a subsample before the initiation of exposure; no specific value is determined | to be measured |
| Feeding regime & starvation period | No feeding during test | No feeding during test | No feeding during test & 24 hr starvation |
| Test concentrations: | Definitive test: minimum of 5 test concentrations chosen in a geometric series plus a dilution water control and a vehicle (solvent) control, if a vehicle is used | Testing the minimum concentration causing 100% and the maximum concentration causing 0% mortality are **not** mandatory requirements | It would be desirable to include within the concentration range the concentrations at which all test fishes die, and that at which there are no fatalities |
| Temperature: | 12°C for Atlantic salmon, Brook trout, Coho salmon, Rainbow trout. 22°C for Atlantic silverside, Bluegill sunfish, Channel catfish, Common carp, Sheepshead minnow, Inland silverside, Tidewater silverside, Fathead minnow, Guppy, Medaka, Zebrafish   Constant during test within ±2°C | Water temperature is species specific and should not differ by more than 2°C between test vessels or between successive days at any time during the exposure, and should be within the temperature ranges specified for the test species e.g. for zebrafish with a range of 24-28°C, the temperature selected could be 27°C and should not vary more than ± 1°C between test vessels and between successive days while staying in the recommended range of 24–28°C, Rainbow trout should stay between the range of 10-14°C | 21-25°C for most fish, 20-24°C for Carp, 13-17°C for trout |
| Aeration: | None - Not recommended; gentle aeration of test vessels may only be used in cases where the dissolved oxygen levels are in danger of dropping below 60% saturation. In such cases, assurances should be made that the use of aeration does not stress the test organisms; test substance concentrations should be measured during the test; and all treatment and control vessels should be given the same aeration treatment | Aeration can be used provided that it does not lead to a significant loss of test chemical as verified by analytical measurements of test concentrations | As far as there is no significant loss of the test substance, gentle aeration may be applied as required |
| pH | Between 6.0 and 8.5 for freshwater testing; between 7.5 and 8.5 for saltwater testing (constant during test within ±1 pH unit) | Between 6.0 and 8.5 for freshwater testing; (constant during test within ±1 pH unit) | Do not adjust the pH of the test solution & measure at each group |
| Water hardness (as CaCO3) (freshwater tests) | For freshwater: <250 mg/L (preferably <180 mg/L); 40-50 mg/L for testing with metals | Should demonstrate stability - 40-180 mg/L CaCO_3_  Hardness greater than 180 mg/L is allowed for certain species. (3000-7500) | General comment: confirm the quality of the dilution water prior to the study |
| Salinity (saltwater tests) | Selected from a range of 15 to 25 ppt (constant during test within ±2 ppt for selected salinity) | 0-0.2‰ ± 2‰ allowed for freshwater species. And up to 35‰ allowed for saltwater species. | General comment: confirm the quality of the dilution water prior to the study |
| Conductivity | should be measured | ≤10 μS/cm | General comment: confirm the quality of the dilution water prior to the study |
| Total organic carbon (TOC) | ≤2 mg/L | ≤2 mg/L | General comment: confirm the quality of the dilution water prior to the study |
| Chemical oxygen demand (COD) | Should be analysed periodically in the dilution water or at the beginning of the test | ≤5 mg/L | General comment: confirm the quality of the dilution water prior to the study |
| Copper (Cu) | Any heavy metals present in feed should be analysed periodically (acceptable levels are not mentioned) | ≤1 μg/L (Copper can cause fish mortality; LC_50_ fathead minnow: 0.073 mg/L) | General comment: confirm the quality of the dilution water prior to the study |
| Observation time points | At 24, 48, 72, and 96 hours. An observation period at <12 hours is desirable. | To the extent feasibly possible, 2-3 observations should be conducted within the first 24 hours of the study with preferably at least 3 hours between observations. On days 2-4 of the test, all vessels with living fish should be inspected twice per day | At the very least, observe the general condition of test fish at the 24, 48, 72, and 96 hours after the commencement of exposure, and keep records. Record any abnormalities/deaths observed |
| Analytical measurements | Compulsory ±20% nominal - analytical method should be validated before beginning the test | Compulsory ±20% nominal - analytical method should be validated where technically feasible | If the measured values for test substance concentration fluctuate ± 20% or more above the nominal concentration, compute LC_50_ on the basis of the mean measured concentration |
| Observations | Any abnormal behaviour or appearance, and the number of individuals exhibiting these characteristics, should be counted and recorded at the same time as observations of mortality | Mortalities and visible abnormalities in regard to equilibrium, appearance, ventilatory behaviour and swimming behaviour are recorded. If possible, additional clinical signs may be reported (set out in Annex 4, Tables 1 and 2) | n/a |
| Death | Dead fish should be removed from the test vessels at the time of observation | Fish are considered dead if there is no visible movement (e.g. gill movements) and if touching of the caudal peduncle produces no reaction. Mortalities are recorded, and dead fish are removed as soon as they are observed | A fish is considered dead if there is no observable movement (of the gill covers, etc.), and there is there is no response when the tail is touched. Promptly remove dead fish from the test system |
| Endpoints required | Concentration-response curve, slope, no observed effect concentration (NOEC) and LC_50_ based on mortality | LC_50_ Median lethal concentration: The test substance concentration at which 50% of the test animals die during the exposure period | LC_50_ Median lethal concentration: The test substance concentration at which 50% of the test animals die during the exposure period |
